# Altering Sterol Composition Implied That Cholesterol Is Not Physiologically Associated With Diosgenin Biosynthesis in *Trigonella foenum-graecum*

**DOI:** 10.3389/fpls.2021.741604

**Published:** 2021-10-18

**Authors:** Liyang Cao, Zilin Zhou, Jia Sun, Changfu Li, Yansheng Zhang

**Affiliations:** ^1^Shanghai Key Laboratory of Bio-Energy Crops, Research Center for Natural Products, Plant Science Center, School of Life Sciences, Shanghai University, Shanghai, China; ^2^CAS Key Laboratory of Plant Germplasm Enhancement and Specialty Agriculture, Wuhan Botanical Garden, Chinese Academy of Sciences, Wuhan, China; ^3^Patent Examination Cooperation (Henan) Center of the Patent Office, China National Intellectual Property Administration, Zhengzhou, China

**Keywords:** diosgenin biosynthesis, sterol origin, sterol 24-methyltransferase 1, cholesterol, *Trigonella foenum-graecum*

## Abstract

Diosgenin serves as an important precursor of most steroidal drugs in market. Cholesterol was previously deemed as a sterol origin leading to diosgenin biosynthesis. This study reports that cholesterol is not in parallel with diosgenin biosynthesis in *Trigonella foenum-graecum*. We first perturbed its sterol composition using inhibitors specific for the upstream isoprenoid pathway enzymes, HMGR (3-hydroxy-3-methylgutaryl-CoA reductase) on the mevalonate (MVA) and DXR (1-deoxy-D-xylulose-5-phosphate reductoisomerase) on the 2-C-methyl-D-erythritol-4-phophate (MEP) pathways, and have revealed that diosgenin and cholesterol reversely or differently accumulated in either the MVA or the MEP pathway-suppressed plants, challenging the previously proposed role of cholesterol in diosgenin biosynthesis. To further investigate this, we altered the sterol composition by suppressing and overexpressing the 24-sterol methyltransferase type 1 (SMT1) gene in *T. foenum-graecum*, as SMT1 acts in the first committed step of diverting the carbon flux of cholesterol toward biosynthesis of 24-alkyl sterols. Knockdown of *TfSMT1* expression led to increased cholesterol level but caused a large reduction of diosgenin. Diosgenin was increased upon the *TfSMT1*-overexpressing, which, however, did not significantly affect cholesterol biosynthesis. These data consistently supported that diosgenin biosynthesis in *T. foenum-graecum* is not associated with cholesterol. Rather, campesterol, a 24-alkyl sterol, was indicative of being correlative to diosgenin biosynthesis in *T. foenum-graecum*.

## Introduction

*Trigonella foenum-graecum*, also named fenugreek, originates from the Iran and Mediterranean regions (Mostafaie et al., 2018). This genus has been the subject of extensive phytochemical investigations for decades (Jain et al., 1996), following the discovery of an important steroid sapogenin, called diosgenin, in it (Fazli and Hardman, 1968). Diosgenin is of great importance mostly because that it serves as a starting precursor used in the pharmaceutical industry for synthesis of more than two hundred steroidal drugs in market (Chifflet, 1945; Fernandes et al., 2003), and this compound itself also shows various antitumor activities. For example, many *in vitro* experiments showed evidence that diosgenin suppressed the proliferation of a variety of cancer cells through modulating multiple signaling pathways (Sethi et al., 2018). In addition, in an intact animal mode, diosgenin was shown to inhibit the growth of rat colon tumor (Raju et al., 2004), human breast cancer (Srinivasan et al., 2009), and lung adenocarcinoma tumors (Yan et al., 2009). Those studies raise the prospect of developing diosgenin as a natural product-based antitumor drug. In contrast with the intense interests on its pharmacological activities, knowledge about the diosgenin biosynthetic pathway is much less established.

**Figure 1:**
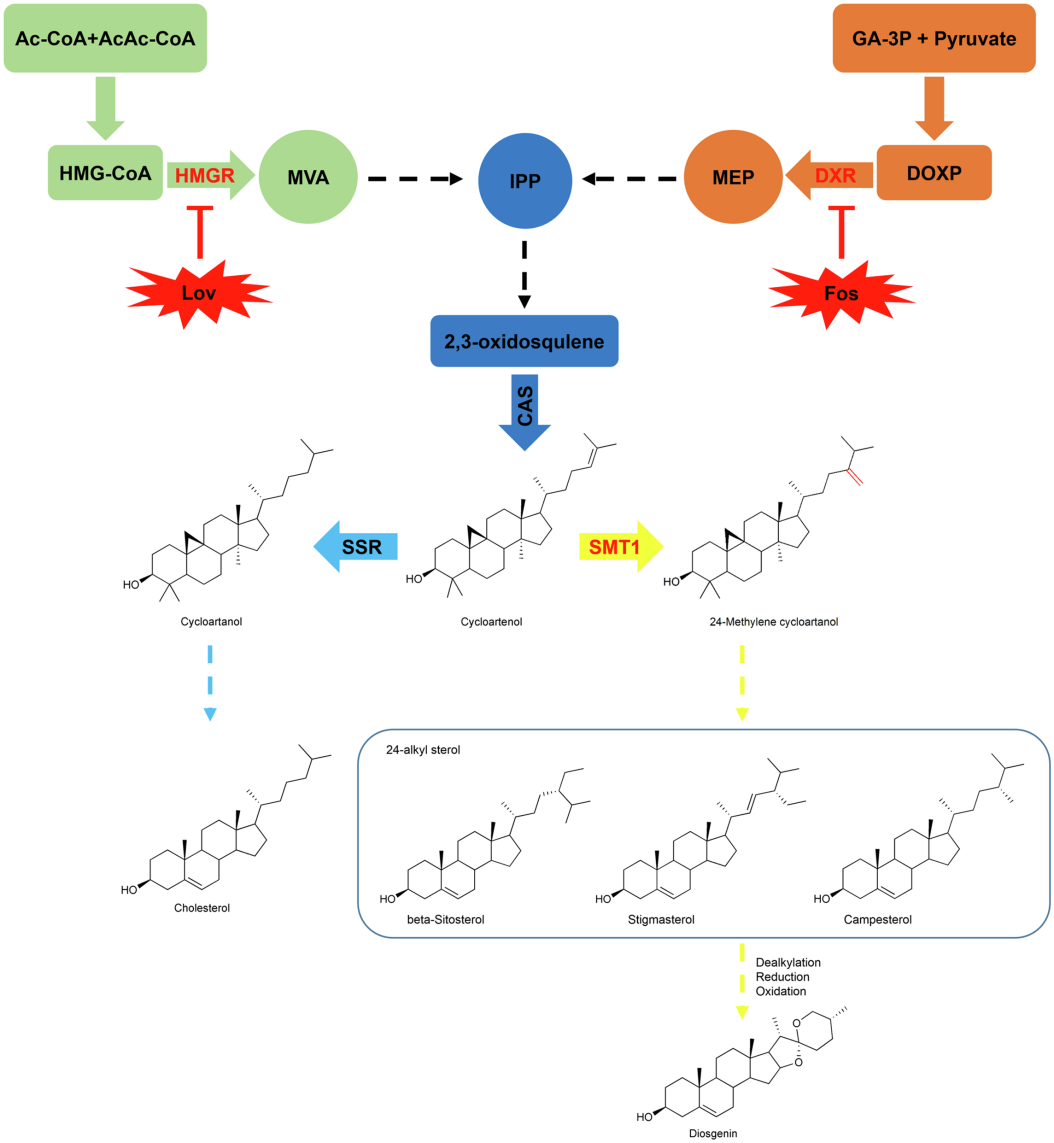
Proposed pathway of diosgenin *via* 24-alkylated sterols. Cholesterol and 24-alkylated sterols are biosynthesized from a common substrate cycloartenol, which is diverted to cycloartanol by SSR (sterol side chain reductase) in the steps up to cholesterol or to 24-methylenecycloartenol by SMT1 (sterol methyltransferase type 1) in the steps toward biosynthesis of 24-alkyl sterols (e.g., stigmasterol, beta-sitosterol, and campesterol). In the upstream steps, they originate from the isoprenoid unit IPP (Isopentenyl diphosphate) *via* the mevalonate (MVA) or the non-mevalonate (MEP) pathway. Lovastatin (Lov) and Fosmidomycin (Fos) are the specific enzyme inhibitors for HMGR (3-hydroxy-3-methylgutaryl-CoA reductase) on the MVA and DXR (1-deoxy-D-xylulose-5-phosphate reductoisomerase) on the MEP pathway, respectively. The activities of dealkylation, oxidation, and reduction presumably involve in the steps from 24-alkylated sterols to diosgenin.

The biosynthesis of diosgenin undoubtedly proceeds *via* the 2,3-oxidosqualene biosynthesized from the isoprenoid pathway. Isopentenyl diphosphate (IPP) and its isomer, dimethylallyl diphosphate (DMAPP), are two common C5-isoprene blocks *via* from the mevalonate (MVA) and 2-C-methyl-D-erythritol-4-phophate (MEP) pathways (Figure 1). HMGR (3-hydroxy-3-methylgutaryl-CoA reductase) and DXR (1-deoxy-D-xylulose-5-phosphate reductoisomerase) are the key enzymes that control the carbon flux through the MVA and MEP pathways, respectively (Carretero-Paulet et al., 2006; Nieto et al., 2009). The 2,3-oxidosqualene is cyclized to yield cycloartenol by cycloartenol synthase (CAS), and cloning of the corresponding *CAS* cDNA from *T. foenum-graecum* was previously reported (Liu et al., 2019). Cycloartenol represents the key branching point leading to biosynthesis of either cholesterol catalyzed by sterol side chain reductase (SSR) or 24-alkyl sterols initiated by 24-sterol methyltransferase type 1 (SMT1; Figure 1; Sonawane et al., 2016). Elevated cholesterol level was observed in *Arabidopsis smt1* mutants (Carland et al., 2010) and a reduced cholesterol level was observed in transgenic potato plants that overexpressed a soybean *SMT1* gene (Arnqvist et al., 2003). Early labeling studies on the seedlings or the undifferentiated cells of the *Dioscorea* species showed that cholesterol can be metabolized to diosgenin in plants (Bennett and Heftmann, 1965; Joly et al., 1969; Stohs et al., 1969). Very recently, Weng et al. isolated relevant cytochrome P450s-encoding genes from *T. foenum-graecum* and *Paris polyphylla*, and showed data that the expressed P450s, when coupled appropriately, catalyze multiple step-oxidations of cholesterol to yield diosgenin in a heterologous expression yeast system (Christ et al., 2019). These observations suggest that cholesterol may act as a direct precursor to diosgenin biosynthesis in plants. However, although cholesterol makes up a large fraction of the total sterol contents in some plant species, such as *Canola*, *Solanaceae*, *Liliaceae*, *Scrophulariaceae*, and *Cucurbita maxima* (Garg and Nes, 1984; Garg et al., 1987; Garg and Nes, 1987; Behrman and Gopalan, 2005), it is indeed a minor sterol in the several diosgenin-producing species with its concentration usually being much lower than that of diosgenin by orders of magnitude (Cerdon et al., 1995; Sun et al., 2017). On the other hand, 24-alkyl sterols, such as β-sitosterol, are biosynthesized at comparable levels with diosgenin *in vivo* (Cerdon et al., 1995; Sun et al., 2017). These findings challenge the precursor role of cholesterol in diosgenin biosynthesis in plants. Comparative transcriptome analysis on different tissues of *Asparagus racemosus* (Upadhyay et al., 2014) and on different varieties of *T. foenum-graecum* with contrasting levels of diosgenin (Chaudhary et al., 2015) argued that β-sitosterol may function as a precursor for diosgenin biosynthesis in plants. Therefore, the sterol origin for diosgenin biosynthesis *in vivo* remains elusive.

The aim of the present research was to provide insights into the sterol origin for diosgenin biosynthesis in *T. foenum-graecum*, we chosen the *T. foenum-graecum*, because that a highly efficient hairy root transformation system was recently available with this species (Garagounis et al., 2020). To this end, the sterol composition of *T. foenum-graecum* was firstly disturbed by treating the plants *in vitro* with specific inhibitors targeted for HMGR on the MVA or DXR on the MEP route, or by genetically modulating the *SMT1* expression *in vivo*. Then, we compared the variations in cholesterol and diosgenin biosyntheses in these sterol composition-perturbed plants. Given that all the targeted enzymes (HMGR, DXR, and SMT1) lie far upstream of cholesterol and diosgenin (Figure 1), if cholesterol is a precursor of diosgenin, then one would expect that both compounds may display a concurrent pattern in response to the sterol altering, at least in one of these treatments. Interestingly, all the sterol-disturbed treatments of this study do not support this premise, indicating that cholesterol may be not physiologically related to diosgenin biosynthesis in *T. foenum-graecum*. Rather, our data favored that campesterol, a 24-alkyl sterol, is more correlative to diosgenin biosynthesis in *T. foenum-graecum*.

## Article Types

Original Research.

## Materials and Methods

### Inhibitor Treatment

Stock solutions of lovastatin (100mM) dissolved in DMSO and fosmidomycin (160mM) in sterilized water were filter sterilized and stored at 4°C. When ready for the experiment, aliquots of stock solutions were added into MS medium to yield the working solution at a final concentration of 100μm for lovastatin and 160μm for fosmidomycin. Seeds of *T. foenum-graecum* were sterilized and germinated as previously described (Carretero-Paulet et al., 2006), and two-day-old seedlings with similar growth sizes were selected for the treatment. In order to normalize variation between individuals, each replicate sample contained groups of 30 seedlings that were incubated in the working solutions where sterilized glass beads were used to fix the seedlings. Plants exposed to DMSO (0.1%, v/v) in the MS medium served as controls of the lovastatin treatments, and the ones incubated in the pure MS medium were used as corresponding controls for the Fos-treated plants. At different time intervals (12, 24, 48, 72, 96,120, and 144h) during the treatment, the whole *T. foenum-graecum* seedlings were harvested for the metabolite analysis and phenotype observation.

### Molecular Cloning and Biochemical Assays of *TfSMT1*

Blast analysis of our previously reported *T. foenum-graecum* transcriptome (Zhou et al., 2019) led to the identification of two *SMT1* candidates (*TfSMT1-1* and *TfSMT1-2*). Their open reading frames (ORFs) were amplified using the primers TfSMT1-1-F/R and TfSMT1-2-F/R from *T. foenum-graecum* cDNA and inserted into a yeast expression vector pESC-URA under the control of the *GAL10* promoter. To supply the cycloartenol substrate for TfSMT1, the cDNA encoding *Arabidopsis thaliana* cycloartenol synthase (*AtCAS*) was isolated *via* RT-PCRs using the primers AtCAS-F/R and cloned into the pESC-TRP expression vector under the control of *GAL1* promoter. Primers used in this work are shown in Supplementary Table 1.

To characterize their biochemical functions, both *TfSMT1-1* and *TfSMT1-2* were separately co-expressed with *AtCAS* by co-transferring their expression vectors into the *WAT11* yeast strain (Pompon et al., 1996; Urban et al., 1997). Transgenic yeasts bearing the empty vectors or expressing the *AtCAS* alone served as the controls. All the yeast cultivations were performed at 30°C and 220rpm. To induce expression of the transformed genes, transgenic yeast cells were cultured in an appropriate dropout medium containing 2% glucose, harvested by centrifugation, and washed three times in sterile water. Cells were then re-suspended to an OD_600_ of 0.8 in an induction medium supplemented with 2% galactose. Approximately 48h after induction, cells were harvested by centrifugation for the product analysis. To extract products from the yeast, yeast cells were re-suspended in sterile water and broken with 0.45-mm glass beads (sigma, acid washed). The lysates were then extracted three times with hexane. The hexane extracts were pooled and dried under vacuum. The residue was subsequently trimethylsilylated with 50μl of 1% TMCS in BSTFA (Regis Technologies Inc., US) for 1h at 80°C, followed by addition of 200μl dichloromethane prior to GC-MS analysis.

GC-MS analysis was performed in a Shimadzu QP-2010-Plus GC machine equipped with a DB-5MS column (0.25mm, 30m, and 0.25μm). One microliter of each sample was injected in a splitless mode at a flow rate of 1ml/min with helium as the carrier gas. The initial temperature was kept at 60°C for 1min, ramped to 300°C at a rate of 30°C/min, and held at 300°C for 15min. The ionization temperature was set at 220°C, and full mass spectra were generated by scanning the *m/z* range from 50 to 550.

### Binary Vector Construction and Hairy Root Transformation

To prepare the plant overexpression construct of *TfSMT1-1*, the *TfSMT1-1* coding sequence was amplified using primers of Ox-TfSMT1-1-F and Ox-TfSMT1-1-R. The *TfSMT1-1* coding region was introduced into pDONR201, followed by LR reaction into the destination vector pK7WG2D by Gateway technology. The RNAi vector, named pK7GWIWG2_II-RedRoot, was used to silence the *TfSMT1* transcripts. A 438-bp coding region of *TfSMT1* was amplified by PCR using the primers RNAi-TfSMT1-1-F/R. The PCR products were introduced into pDONR201 and then cloned by recombination into the pK7GWIWG2_II-RedRoot. The resultant plant expression constructs, as well as their respective empty vectors as negative controls, were transformed into *Agrobacterium rhizogenes* ARqua1 (Boisson-Dernier et al., 2001) by electroporation. The hairy roots were generated following the previously described methods (Garagounis et al., 2020) with some modifications. In brief, *T. foenum-graecum* seeds were disinfected with 70% ethanol for 1min and rinsed three times with sterilized water. They were then sterilized in 10% NaClO for 10min and washed with sterilized water for 3–4 times. The sterilized seeds were subjected to germination on 0.65% (w/v) agar plates in darkness at 24°C for 36h, and the geminated seedlings were then used for transformation. The seedling radicles were cut and the traumatized radicle surface was dipped in the *Agrobacterium* slurry harboring the target vectors for 3–5min. The inoculated seedlings were transferred to large square petri dishes containing solid half-strength MS medium. The dishes were placed in a 22°C-growth chamber (16h light/8h dark cycle), first at an angle of approximately 45 degrees for 36h, and then in near vertical. Calluses formed on the infected sites about 2weeks after inoculation, and hairy roots started to emerge about 3weeks post the infection. About 50–60days later, the positive roots were confirmed by GFP or RFP fluorescence examination and used for the metabolite and gene expression analysis. Each biological replicate contained the hairy roots that were pooled from 30 infected plants, and data for each construct were collected from at least three biological repeats.

### Sterol Composition Analysis

Hairy roots were powdered in liquid nitrogen and dried at 37°C. The extraction was carried out as follows: 15mg of each dried hairy root sample was subjected to extraction with 2ml of KOH/MeOH (6%, w/v) for 4h at 80°C and then diluted with equal volume of water. The lysates were extracted three times with 3ml of hexane. The hexane extracts were pooled, washed by sterile water, and dried under vacuum. The residue was dissolved in 50μl of 1% TMCS in BSTFA and incubated at 80°C for 1h. After addition of 100μl of dichloromethane, the derivatized samples were subjected to GC-MS analysis as mentioned above. Ergosterol was used as an internal standard to normalize possible variations introduced from different extractions.

### Diosgenin Analysis

Every 15mg of dried hairy roots was extracted with 1.5ml of methanol for three times, and the methanol extracts were pooled, dried under fume hood, and lysed with 3ml of 1.8M sulfuric acid at 96°C for 8h. The acid-hydrolyzed sample was then extracted three times with hexane. The hexane extracts were washed with sterile water, evaporated to dryness, and dissolved in 150μl methanol for HPLC (High Performance Liquid Chromatography) analysis. To monitor the variation in extraction efficiency between samples, ursolic acid was used as an internal standard.

HPLC analysis was performed on an LC-20AT instrument (Shimadzu, Kyoto, Japan) using an inertsil ODS-SP reverse-phase column (250mm×4.6mm, 5μm). The column temperature was set at 37°C. For analysis of the root extracts, 0.1% phosphoric acid (solvent A) and HPLC-grade methanol/acetonitrile (v/v=7:3; solvent B) were used as the mobile phase, and samples were separated in A:B (10:90) at a flow rate of 0.8mlmin^−1^ for 45min. The detection wavelength was set at 203nm.

### Quantitative Real-Time PCR

Total RNAs were isolated from the hairy root samples using EASYspin Plus Plant RNA Kit (Aidlab, Hangzhou, China). First-strand cDNA was prepared using a First-Strand cDNA Synthesis Kit (Thermo Fisher). The *T. foenum-graecum* actin gene was used as an internal standard to normalize the variation in each cDNA preparation. The qRT-PCR was performed on an ABI 7500 Fast Real-Time PCR Detection System with FastStart Universal SYBR Green Master mix (Rox; Roche, Mannheim, Germany). The PCR conditions were as follows: 10min of initial denaturation at 95°C, followed by 40cycles of 95°C for 15s and then 60°C for 1min. All the RT-PCRs were performed in three biological repeats. Gene specific primers used for the qRT-PCRs are listed in Supplementary Table 1.

### Statistical Analysis

Every experiment was carried out at least in three biological replicates, and data were shown as mean±SD. Data analysis was performed by one-way ANOVA. Difference was considered statistically significant when ^**^p < 0.05 and extreme significant when ^***^p < 0.01.

## Results

### Two Specific Inhibitors Differentially Altered Cholesterol and Diosgenin Accumulations in *T. foenum-graecum*

**Figure 2:**
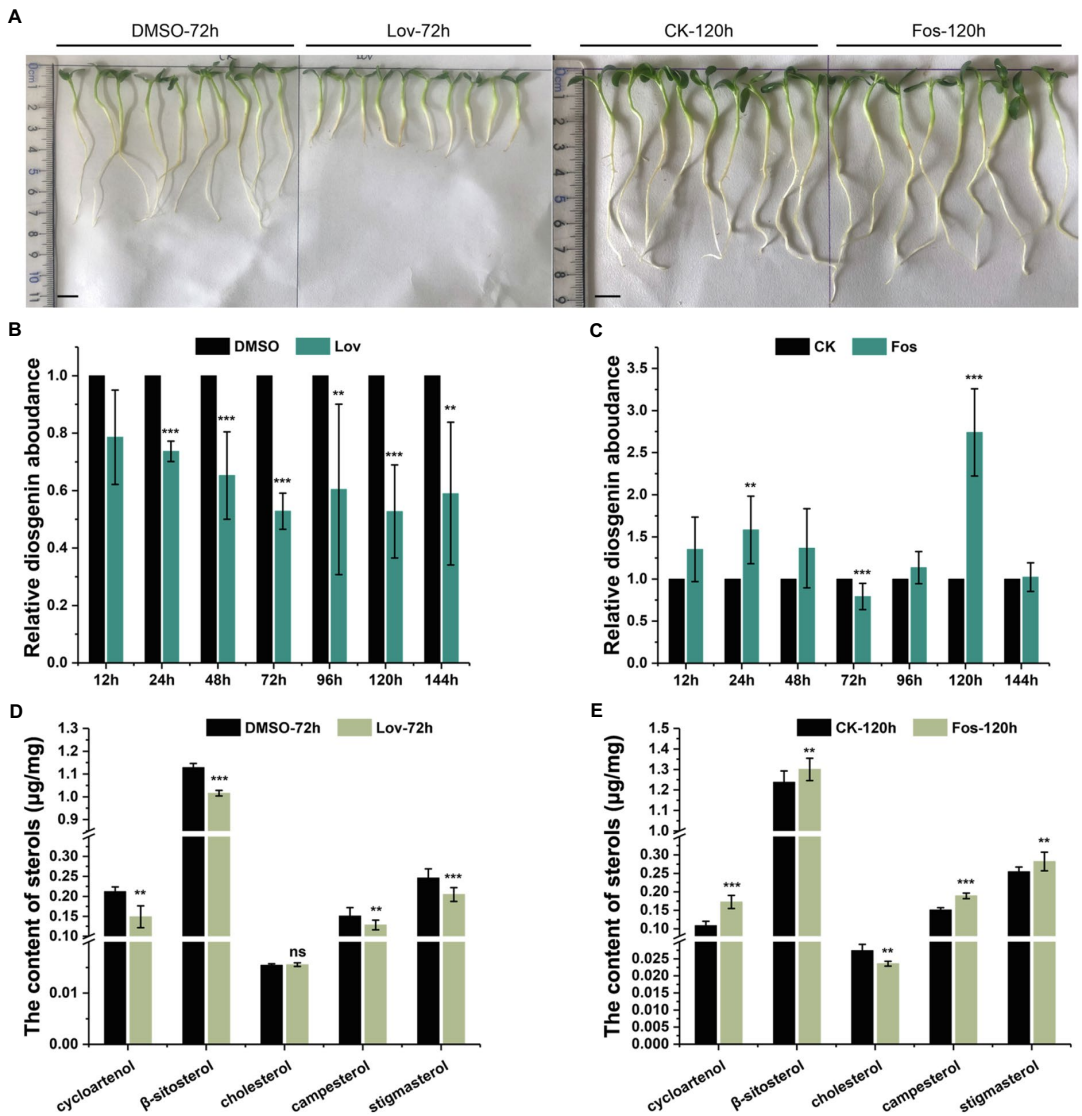
The effects of Lov- and Fos-treatments on the root growth, and accumulation of diosgenin and several sterols of interest in *T. foenum-graecum* plants. **(A)**, representative growth phenotype of 2-day-old *T. foenum-graecum* seedlings treated by 100μm lovastatin (Lov) and the solvent control (DMSO) for 72h, and 160-μm fosmidomycin (Fos) and its corresponding blank control for 120h. Scale bar=1cm. **(B)** and **(C)**, the effects of Lov- and Fos-treatment on diosgenin accumulation in *T. foenum-graecum* seedlings under different time points. Diosgenin level of each time point control was considered as a value of 100%, and relative diosgenin abundance in the Lov- or Fos-treated plants was expressed as ratios to that of the control. **(D)** and **(E)**, the effects of Lov-72-h- and Fos-120 -h-treatments on the accumulation of cycloartenol, cholesterol, and several 24-alkyl sterols (β-sitosterol, campesterol, and stigmasterol). Data represent the averages of three biological repeats and error bars designate s.d. (^**^p < 0.05 and ^***^p < 0.01; ns, not significant by one-way ANOVA).

Lovastatin (Lov) is an effective compound that specifically inhibits the HMGR activity on the MVA route (Xiong et al., 2019), and fosmidomycin (Fos) specifically inhibits the MEP pathway primarily *via* inhibition of DXR (Zeidler et al., 1998). Two-day-old *T. foenum-graecum* seedlings were subjected to the Lov- and Fos-treatments for different time lengths (see details in Method and Material section), and the treated seedlings were analyzed for altered diosgenin level and sterol composition. The Fos-treatments did not lead to an altered visual phenotype compared to the controls at all the time points, whereas a stunted root growth phenotype started to emerge after 72h of the Lov-treatments (see a representative phenotype as shown in Figure 2A).

For comparison of diosgenin level, diosgenin level in the controls of each time point was considered as a value of 100%, and relative diosgenin abundance in the Lov- or Fos-treated plants was expressed as ratios to that of controls. A decrease in diosgenin level was observed in all the Lov-treated plants, particularly about 50% of the reduction occurred after the 72-h incubation (Figure 2B). Conversely, fosmidomycin generally increased the diosgenin production to different extents over the controls at most of the time points, with the exception of a decrease of diosgenin observed at the 72h and no significant changes seen at the 144h (Figure 2C). Especially, the level of increase in diosgenin was about 170% at the 120-h time point (Figure 2C). Subsequently, the Lov-72-h- and Fos-120-h-treated samples, as well as their corresponding controls, were selected for the analyses on sterol composition, including cholesterol, β-sitosterol, stigmasterol, campesterol, and cycloartenol. Among the selected sterols, cholesterol differentiates from others by absence of an alkyl-group on C-24 position (see their structures in Figure 1). Compared with the control, the Lov 72-h-treated plants accumulated a substantially less amount of campesterol and cycloartenol, and slightly decreased β-sitosterol and stigmasterol, whereas cholesterol level was not significantly altered (Figure 2D). In contrast, the Fos 120-h-treatment largely decreased cholesterol biosynthesis but elevated levels of all the tested 24-alkyl sterols (Figure 2E).

### Sterol Composition and Diosgenin Accumulation Altered by Modulating *SMT1* Expression *in vivo*

The data described above indicated that diosgenin biosynthesis in *T. foenum-graecum* seemed to be more associated with 24-alkylated sterols than with cholesterol, a sterol that was previously suggested to be a natural intermediate during diosgenin biosynthesis in plants (Bennett and Heftmann, 1965; Joly et al., 1969). SMT1 catalyzes the first committed step in diverting cycloartenol toward biosynthesis of 24-alkyl sterols (Figure 1). To further investigate a possible role of 24-alkyl sterols in diosgenin biosynthesis, we carried out cloning of *SMT1* from *T. foenum-graecum* (*TfSMT1*), altered its expression by overexpression or RNAi (RNA interference) strategy, and investigated the effects of the transgene events on the sterol composition and diosgenin biosynthesis in *T. foenum-graecum*.

**Figure 3:**
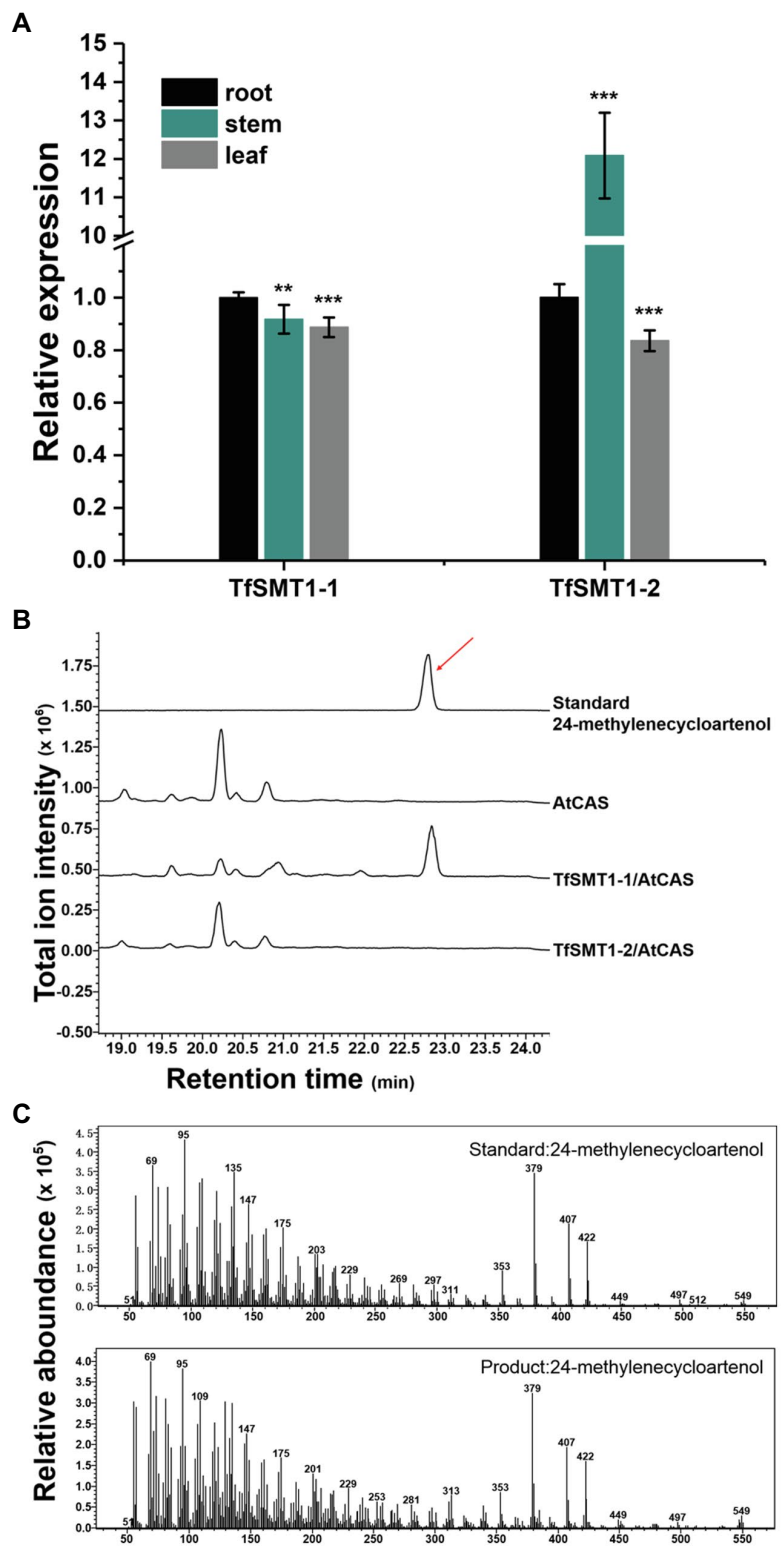
Gene expression patterns in different tissues and biochemical functions of TfSMT1-1 and TsSMT1-2. **(A)**, relative transcript abundances of *TfSMT1-1* and *TfSMT1-2* gene in different tissues (root, shoot, and leaf) analyzed by real-time PCR. The gene expression level in the root organ was considered as a value of 100% and that in other tissues was accordingly normalized (*n*=3 biological replicates, ^**^p < 0.05 and ^***^p < 0.01 by one-way ANOVA). **(B)**, *in vivo* functional assays of TfSMT1-1 and TfSMT1-2 co-expressed with the *Arabidopsis thaliana* cycloartenol synthase (AtCAS) in yeast. GC profile was shown for the products from the yeast cell expressing TfSMT1-1/AtCAS, TfSMT1-2/AtCAS, and AtCAS alone. **(C)**, the mass spectra of 24-methylenecycloartanol standard and the 24-methylenecycloartanol product formed by TfSMT1-1.

Our previously constructed *T. foenum-graecum* transcriptome (Zhou et al., 2019) was used to search for the *SMT1* gene(s), identifying two isoform candidates (designated *TfSMT1-1* and *TfSMT1-2*). TfSMT1-1 has an N-terminal extension of 33 amino acid residues compared to TfSMT1-2 (Supplementary Figure 1). Gene expression analysis showed that the *TfSMT1-1* shows an almost even transcript distribution pattern in the root, stem, and leaf tissues, whereas the *TfSMT1-2* is highly expressed in the stem (Figure 3A). The catalytic functions of TfSMT1-1 and TfSMT1-2 were then investigated by co-expressing them in the yeast WAT11 strain (Urban et al., 1997) with an *Arabidopsis thaliana* cycloartenol synthase (AtCAS), which supplies cycloartenol as a substrate of SMT1 (Corey et al., 1993). A new product was detected when extracts of the yeast strain expressing TfSMT1-1 were analyzed by gas chromatography-mass spectrometry (GC-MS; Figure 3B). This product was identical to the authentic 24-methylenecycloartanol in terms of both retention times and mass spectra (Figures 3B,C). No new products were found from the extracts of the yeast strain expressing TfSMT1-2 (Figure 3B), although it is highly expressed in the stem, reflecting a high rate of sterol biosynthesis in the growing stem tissue.

**Figure 4:**
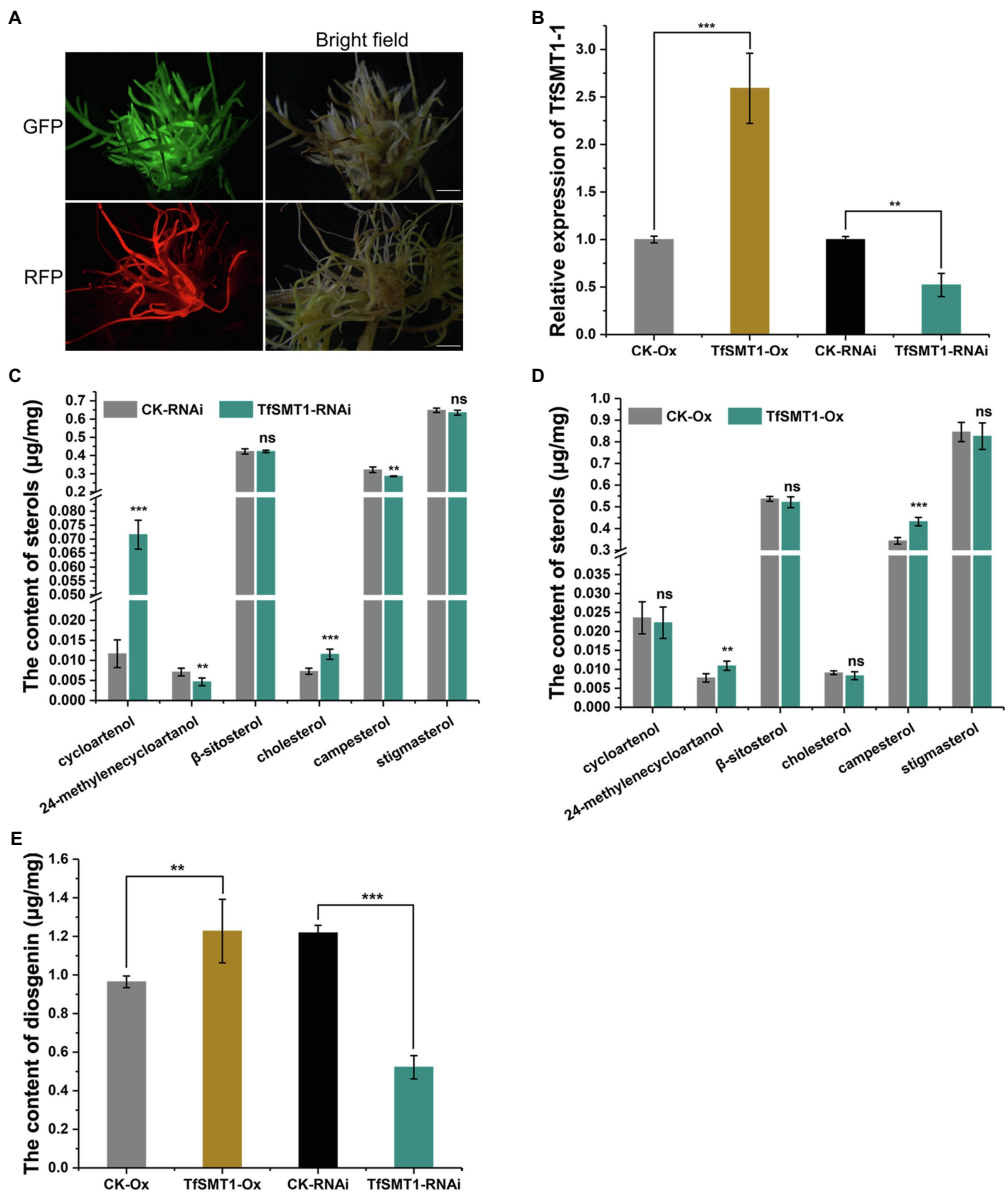
The effects of overexpression and RNAi-based downregulation of *TfSMT1-1* on accumulation of diosgenin and the targeted sterols in the transgenic *T. foenum-graecum* hairy roots. **(A)**, fluorescence signal was shown for the presence of the overexpression TfSMT1-Ox (GFP) and TfSMT1-RNAi (RFP) constructs in the *T. foenum-graecum* hairy roots. Scale bar=1cm. **(B)**, real-time PCR analysis of the *TfSMT1-1* transcripts in the TsSMT1-Ox, TfSMT1-RNAi, and their corresponding empty vector control lines. **(C)** and **(D)**, the contents of the targeted sterols extracted from the hairy roots of TfSMT1-RNAi (C) and TfSMT1-Ox **(D)** lines in comparison with their corresponding controls. **(E)**, the effects of the overexpression and downregulation of *TfSMT1-1* gene on diosgenin biosynthesis in the transgenic hairy roots. All these experiments were performed in three biological replicates. Asterisk indicates a significant difference by one-way ANOVA analysis (^**^p < 0.05; ^***^p < 0.01; and ns, not significant).

To assess the contribution of the flux through the 24-alkyl sterol pathway to diosgenin biosynthesis in *T. foenum-graecum*, we generated the transgenic *T. foenum-graecum* hairy roots where *TfSMT1-1* was overexpressed (*TfSMT1*-Ox) or silenced (*TfSMT1*-RNAi). The control hairy roots were generated by transformation of the respective overexpression or RNAi empty vector. The overexpression vector pK7WG2D that we used contains a GFP (green fluorescent protein) selection marker, and the RNAi binary vector pK7GWIWG2_II-RedRoot contains a RFP (red fluorescent protein) marker. The transgenic roots were screened by fluorescence examination under a microscope for the presence of the transgenes (Figure 4A). The *TfSMT1*-RNAi lines showed an obvious growth-reduction phenotype (Supplementary Figure 2), whereas the *TfSMT1*-Ox lines were phenotypically indistinguishable from the vector control plants (data not shown). As expected, real-time PCR analysis of the transgenic roots showed that expression of the *TfSMT1-1* was substantially higher in the *TfSMT1*-Ox lines and was significantly decreased in the *TfSMT1*-RNAi lines, compared with their, respectively, empty vector-transformed roots (Figure 4B). The effects of the transgene events on the root steroid profiles were assayed by GC-MS analysis. Our data showed that the *TfSMT1*-RNAi roots had significantly lower levels of 24-methylenecycloartanol and campesterol, and higher levels of cycloartenol and cholesterol, as compared to the empty vector controls (Figure 4C), indicating that the suppression of the TfSMT1 activity diverted more carbon flux from cycloartenol toward the cholesterol pathway. Contents of β-sitosterol and stigmasterol (end product sterols) were not affected in the *TfSMT1*-silenced roots as compared to the control lines (Figure 4C). In the *TfSMT1*-Ox lines, 24-methylenecycloartanol and campesterol were increased by 41 and 26%, respectively, while no significant changes were observed for other steroids when compared to the control roots (Figure 4D). Interestingly, downregulation of *TfSMT1* led to a halving of the amount of diosgenin (Figure 4E), albeit with a significant increase of cholesterol (Figure 4C), the sterol previously proposed to be a precursor of diosgenin. Diosgenin also showed a slight increase (about 27%) in the *TfSMT1*-Ox roots, when compared to the control roots (Figure 4E). These data demonstrated that SMT1 plays a positive role in *T. foenum-graecum* diosgenin biosynthesis.

## Discussion

Several previous experiments have shown that exogenously supplied radio-labeled cholesterol can be metabolized to diosgenin in the *Dioscorea* species (Bennett and Heftmann, 1965; Joly et al., 1969; Stohs et al., 1969), seeming to accept cholesterol as an intermediate *en route* to diosgenin. However, those experiments have neglected a fact that the exogenously fed cholesterol may cross physiological membrane barriers to arrive at some specific subcellular sites to which endogenous cholesterol naturally does not have an access, thereby probably introducing unnaturally metabolic fate for cholesterol. This study presents evidence not supporting cholesterol as a biogenetic precursor of diosgenin in *T. foenum-graecum*. First, specific enzyme inhibitors of MVA (lovastatin) and MEP (fosmidomycin) pathways were fed to the intact *T. foenum-graecum* plants, and their effects on the biosyntheses of cholesterol and diosgenin were evaluated. Our data clearly showed that diosgenin biosynthesis was not in parallel with cholesterol in the two specific inhibitors-treated plants. For example, the MVA-suppressed plants had an increased level of cholesterol, whereas the MEP pathway inhibition caused a severe reduction in cholesterol accumulation (Figures 2B,C). This result was in accordance with a previous report (Singh et al., 2014), where expression of DXR from the MEP pathway showed a strongly positive correlation with cholesterol biosynthesis. Moreover, cholesterol showed a reverse accumulation pattern with diosgenin in the inhibitors-mediated changes. For instance, the lovastatin treatment showed reduction in diosgenin accumulation while led to an enhancement in cholesterol (Figure 2B). Conversely, the Fos-treatment decreased cholesterol biosynthesis but increased diosgenin levels (Figure 2C). An increase in diosgenin observed in the Fos-treated plants was not anticipated; however, this increment, probably as a consequence, resulted from the migration of IPP from the MEP to the MVA pathway. Indeed, Laule et al. (Laule et al., 2003) showed that the Fos-treatment facilitated a more pronounced translocation of IPP from plastid to cytoplasm than from cytosol to plastid in *Arabidopsis*. Considering that HMGR or DXR lies far upstream of cholesterol and diosgenin (Figure 1), if cholesterol functions as a precursor of diosgenin, they may display a concurrent accumulation pattern, when the HMGR or the DXR activity is inhibited. Thus, the different or even reverse accumulations in cholesterol and diosgenin in the inhibitors-treated plants convinced us to suggest that cholesterol does not physiologically correlate with diosgenin in *T. foenum-graecum*. When the *T. foenum-graecum* seeds were germinated to the seedlings, a large decrease in cycloartenol accompanied with a significant increase in β-sitosterol, campesterol, or stigmasterol was observed (Supplementary Figure 4), suggesting that the TfSMT1 activity is activated during this developmental process, and at least some of the 24-alkyl sterols are newly synthesized in the seedlings, where although a large fraction of the 24-alkyl sterols may be directly derived from the seeds. The biosyntheses of cycloartenol, cholesterol, and several 24-alkyl sterols (i.e., β-sitosterol, campesterol, and stigmasterol) were monitored in the two inhibitors-treated seedlings. The tested 24-alkyl sterols all exhibit a similar pattern to diosgenin in the both inhibitor-treated plants, as they were in a parallel manner with diosgenin increased by the Fos inhibition and were decreased by the Lov-treatment (Figure 2).

Starting from cycloartenol, SMT1 catalyzes the first committed step in the pathway leading to 24-alkyl sterols (Figure 1). To further provide insights into the precursor origin of diosgenin biosynthesis at a genetic level, we set out to modulate expression of *SMT1* in *T. foenum-graecum in vivo*. Quantitative RT-PCR analysis data showed the success of modulating *SMT1* expression in the present study by overexpression or RNAi technology (Figure 4B). In comparison with the controls, the *TfSMT1*-RNAi lines showed reduced plant size and increased cholesterol content, which seem to be two common features for the *SMT1*-silenced plants or *smt1* mutants. For example, when the SMT1 was suppressed in *Arabidopsis* (Diener et al., 2000), rice (Chen et al., 2018), and *Withania somnifera* (Pal et al., 2019), they all displayed a dwarfism phenotype and increased levels of cholesterol. Therefore, the appearance of reduced plant growth and increased cholesterol content in our *TfSMT1*-RNAi lines indicated that the *TfSMT1* expression was correctly suppressed *T. foenum-graecum*. Once again, by downregulating the *TfSMT1* transcripts, the sterol that showed more close relationship with diosgenin was identified to be campesterol rather than cholesterol, as diosgenin and campesterol were concurrently decreased in the *TfSMT1*-RNAi lines where the cholesterol change trend was the reverse (Figure 4C). These findings demonstrated that TfSMT1 plays an important role in diosgenin biosynthesis in *T. foenum-graecum*. Among the root, stem, and leaf tissues of *T. foenum-graecum*, diosgenin was found to be the highest accumulated in the leaf (Supplementary Figure 3). The same set of tissues was also tested for expression of *TfSMT1* (Figure 3A), and *SSR2* and other three precursor genes (*SS*, squalene synthase; *SE*, squalene epoxidase; and *CAS*, cycloartenol synthase; Supplementary Figure 3). However, none of these upstream genes showed a tissue-expression pattern similar to that for diosgenin accumulation (Supplementary Figure 3). The tissue-specificity of diosgenin accumulation may be governed only by its downstream pathway genes, or it is transported between the tissues from the initial biosynthesis site *in vivo*. Based on the gene expression data acquired by RNA-sequencing of *T. foenum-graecum* seedlings (Zhou et al., 2019), it appeared that the expression of *TfSMT1*, *SS*, and *CAS*, but not *SSR2* and *SE*, was strongly induced by methyl jasmonate (MeJA) which also increased diosgenin biosynthesis (Zhou et al., 2019). Similarly, by a comparatively transcriptomic analysis, SMT1 was indicative of being correlative to diosgenin biosynthesis in *Asparagus racemosus* (Upadhyay et al., 2014). Although the *TfSMT1* expression was largely suppressed in the *TfSMT1*-RNAi lines (Figure 4B), the content of β-sitosterol in it was not significantly affected with respect to controls (Figure 4C). Pal et al. also reported that the levels of β-sitosterol were not significantly altered by *SMT1*-silencing in *Withania somnifera* (Pal et al., 2019). The reduced plant size phenotype of the *TfSMT1*-RNAi lines (Supplementary Figure 2) might be due to reduction in campesterol content (Figure 4C) that caused a low ratio of campesterol to β-sitosterol. Schaller et al. ever reported that a low ratio of campesterol to β-sitosterol was associated with reduced plant growth (Schaller et al., 1998; Schaeffer et al., 2001). On the other hand, in the *TfSMT1-1*-Ox lines, only a slightly increase was observed for campesterol (26%) and diosgenin (27%), whereas other sterols displayed no significant changes in content compared to the controls, suggesting that there was a challenge in boosting the phytosterol pathway through the SMT1 channel. Given that campesterol is generally considered a precursor of C28-brassinosteroids (BRs; Bajguz et al., 2020), and the modulation in campesterol concentrations probably would change the BR levels, then one might argue that the observed variations in diosgenin biosynthesis in the *TfSMT1*-expression altered plants could be due to potential regulations stimulated by the BR changes. However, Nelson et al. previously reported that the deficiency of SMT1 did not change the BR levels in *Arabidopsis* (Carland et al., 2010), and moreover, the defective phenotype of the *SMT* mutants is distinct from that of the BR mutants (Schaller et al., 1998) and different sets of downstream genes are affected by sterol and BR pathway mutants (He et al., 2003). Of course, further experiments are needed to clarify whether a 24-alkyl sterol could form a precursor-product relationship with diosgenin, a non-BR-type sterol, in *T. foenum-graecum*. Indeed, campesterol was ever indicative of playing a precursor role in biosynthesis of withanolide (Pal et al., 2019), which also represents one class of non-BR-type steroids.

Taken together, through perturbing sterol composition of *T. foenum-graecum via* an inhibitor treatment at a physiological level and manipulation of *SMT1* expression at a molecular level, this study has revealed that diosgenin biosynthesis is more associated with campesterol (24-methyl cholesterol) than with cholesterol in *T. foenum-graecum*, although an involvement of cholesterol in diosgenin biosynthesis still could not be completely ruled out. If campesterol indeed participates in diosgenin biosynthesis as a precursor, enzyme(s) responsible for removing a C24-methyl group of campesterol must be involved in the steps from campesterol to diosgenin. Enzymes capable of catalyzing 24-dealkylation activities toward plant 24-alkyl sterols have been isolated from parasitic nematodes (Chitwood and Lusby, 1991); however, they have not been isolated from any plant species so far. Demethylation is a common process in biosynthesis of phytosterols, and, for example, a known CYP51 member catalyzes a sterol C14-demethylating reaction in biosynthesis of cholesterol and 24-alkyl sterols (Sonawane et al., 2016). Indeed, we previously have identified six *T. foenum-graecum* CYP51 candidates whose expressions were stimulated by MeJA, which also enhanced diosgenin accumulation (Carretero-Paulet et al., 2006). From these six P450 candidates, further studies need to be conducted to investigate whether we can reveal the enzyme with such a 24-demethylation activity.

## Conclusion

This study provides a novel insight that cholesterol is not physiologically associated with diosgenin biosynthesis in *T. foenum-graecum*. First, through suppressing the upstream isoprenoid pathway enzymes (i.e., HMGR on the MVA or DXR on the MEP route) in *T. foenum-graecum* by specific inhibitors, followed by measuring their effects on the biosyntheses of cholesterol and diosgenin, we have found that diosgenin was not accumulated in a parallel manner with cholesterol. Next, we perturbed the biosynthesis of cholesterol in *T. foenum-graecum* by genetically altering the *TfSMT1* expression and have found that the knockdown of *TfSMT1* expression significantly increased cholesterol biosynthesis as expected, whereas this transgene resulted in a large decrease in diosgenin. We discussed that a 24-alkylated sterol, campesterol, may play a role in diosgenin biosynthesis.

## Data Availability Statement

The original contributions presented in the study are included in the article/Supplementary Material, and further inquiries can be directed to the corresponding author.

## Author Contributions

YZ designed the experiment and wrote this manuscript. LC, ZZ, and JS performed the experiments. CL analyzed the data. All authors read and confirmed the content.

## Funding

The work was jointly supported by a grant from the National Key R&D Program of China (2018YFC1706200) and a grant from the National Natural Science Foundation of China (31670300).

## Conflict of Interest

The authors declare that the research was conducted in the absence of any commercial or financial relationships that could be construed as a potential conflict of interest.

## Publisher’s Note

All claims expressed in this article are solely those of the authors and do not necessarily represent those of their affiliated organizations, or those of the publisher, the editors and the reviewers. Any product that may be evaluated in this article, or claim that may be made by its manufacturer, is not guaranteed or endorsed by the publisher.
