# Supplementary material for: Altering Sterol Composition Implied That Cholesterol Is Not Physiologically Associated With Diosgenin Biosynthesis in Trigonella foenum-graecum
Source: Front Plant Sci. 2021 Oct 18;12:741604. doi: 10.3389/fpls.2021.741604 (PMC8558557; doi:10.3389/fpls.2021.741604)
Supplement: Supplementary file 1 [file Data_Sheet_1.DOCX]

Supplementary Material

## Supplementary Figures


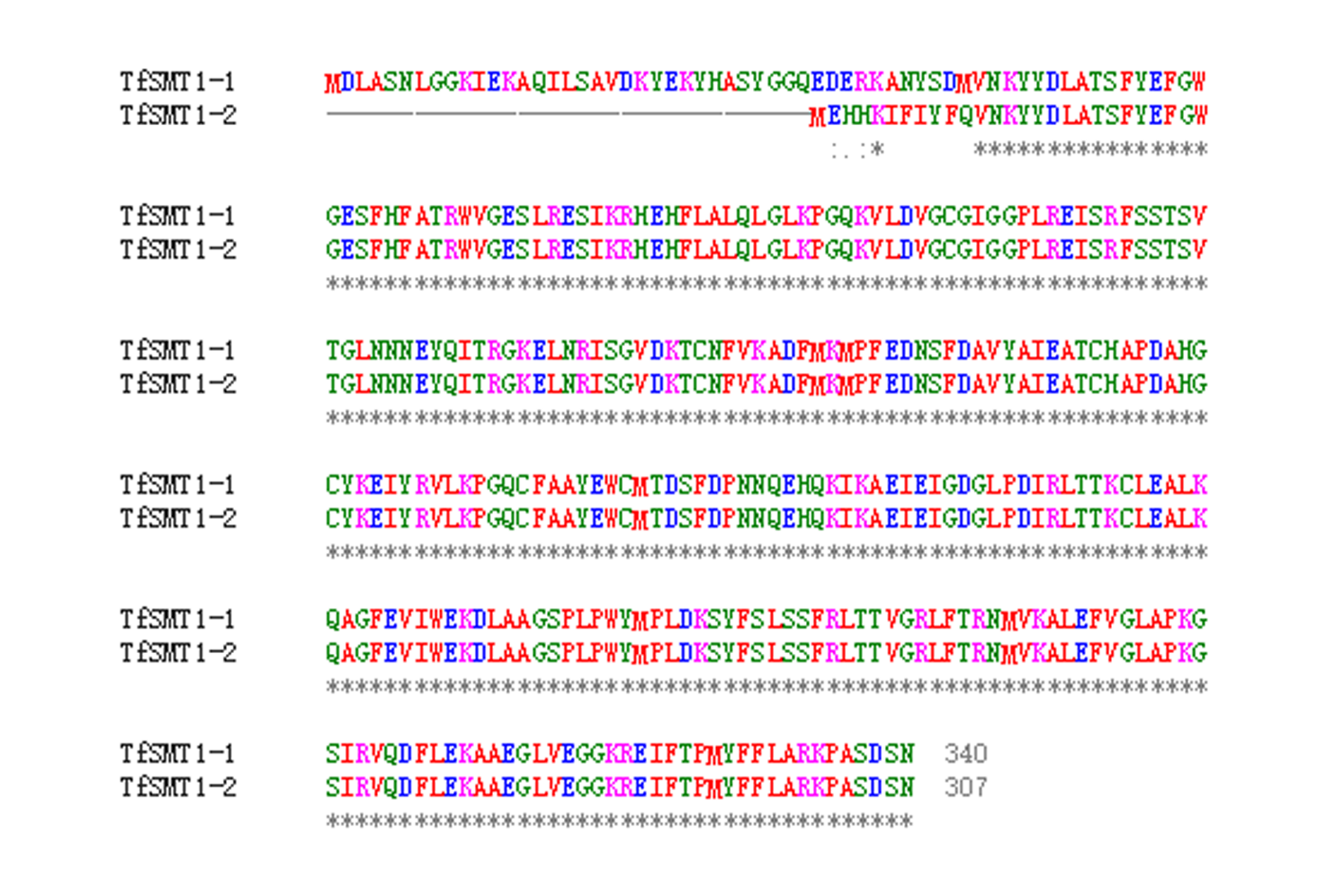


**Supplementary Figure 1.** Alignment of amino acid sequences of TfSMT1-1 and TfSMT1-2. TfSMT1-1 has an N-terminal extension of 33 amino acid residues compared to TfSMT1-2. TfSMT1-1 and TfSMT1-2 differ by 44-amino acids in their N-terminus.


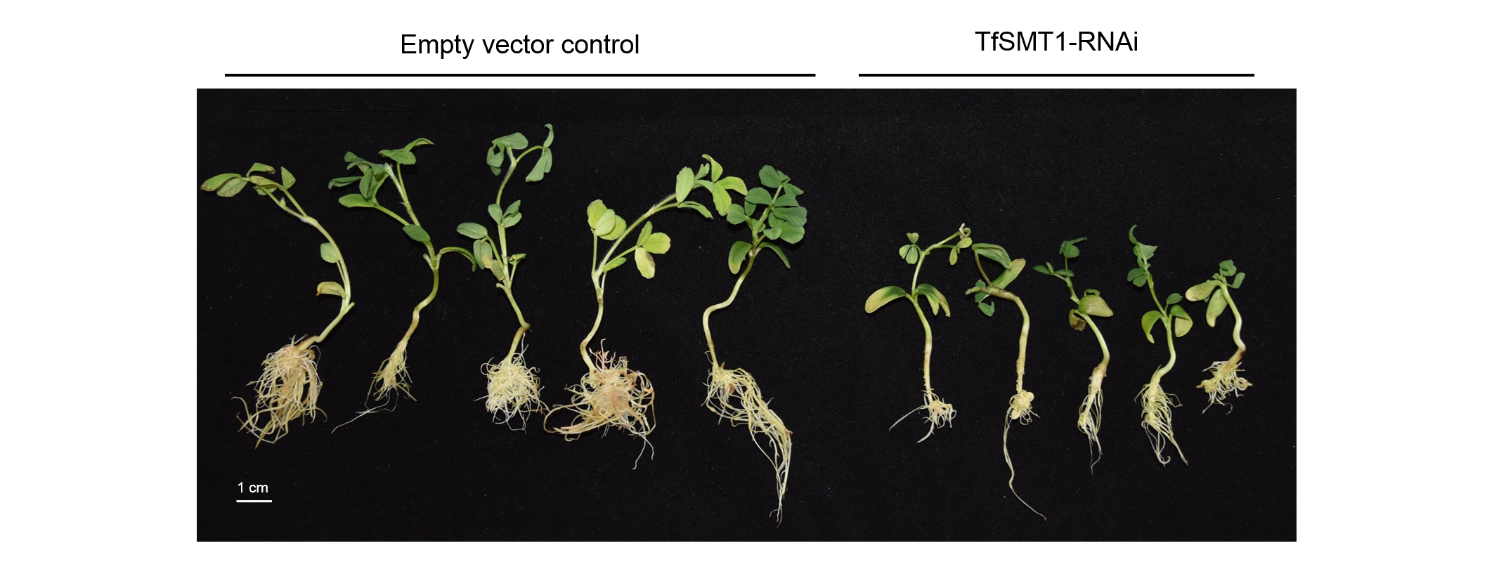


**Supplementary Figure 2.** The representative growth phenotype of TfSMT1-RNAi seedlings compared with and the respective empty vector controls. The TfSMT1-RNAi lines showed an obviously growth-reduction phenotype compared to the controls. Scale bar = 1 cm.


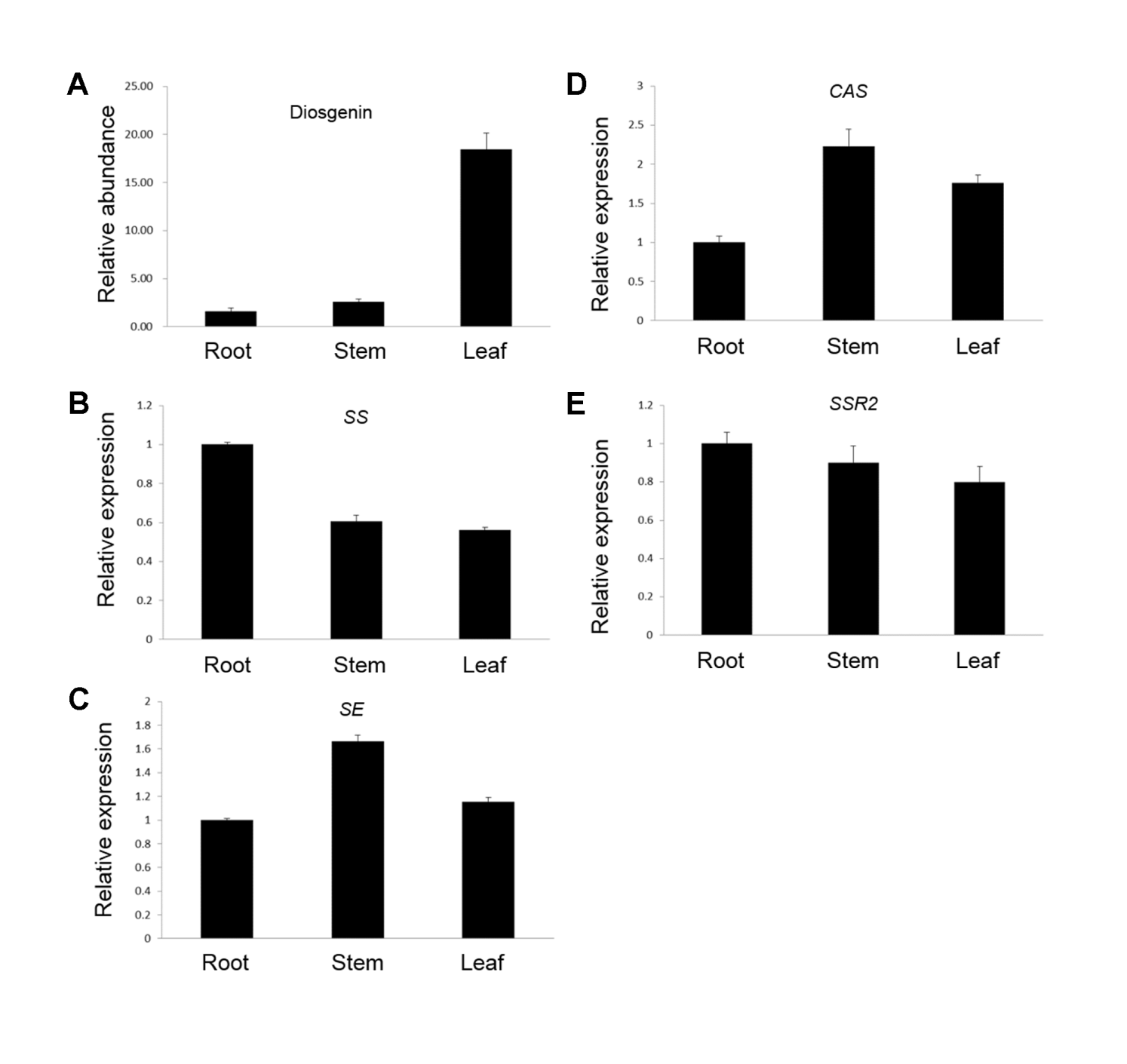


**Supplementary Figure 3.** Diosgenin accumulation and the expression patterns of several upstream genes (*SS*, *CAS*, *SE*, and *SSR2*) in different tissues of *T. foenum-graecum.*


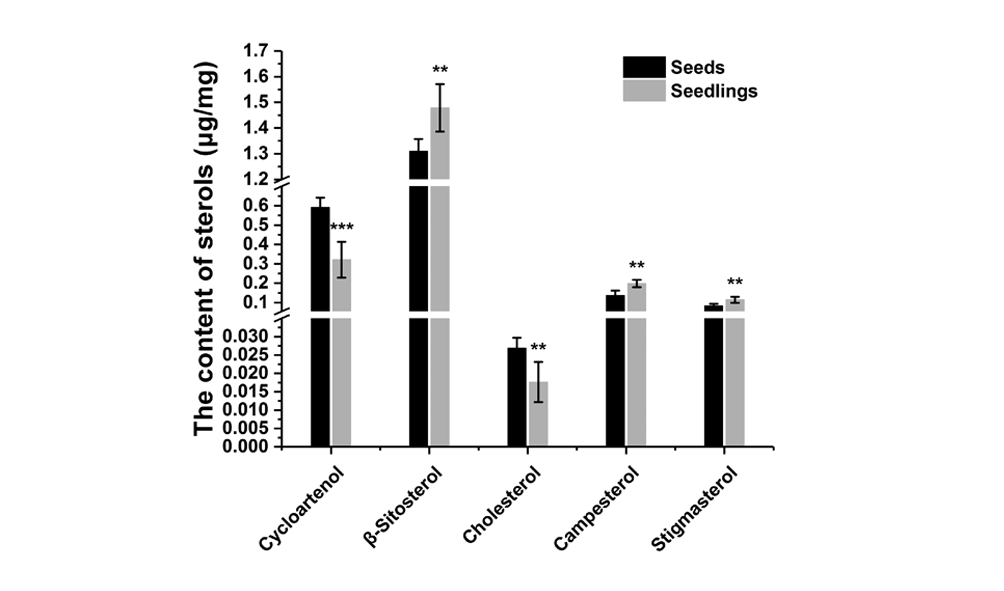


**Supplementary Figure 4.** Comparison of the sterol contents between the *T. foenum-graecum* seeds and the 36 h-germinated seedlings*.*

## Supplementary Tables

Supplementary Table 1. Primers used in this study

| **Primer name** | **Sequence (5' to 3')** | **Description** |
| --- | --- | --- |
| TfSMT1-1-F | GAATTCatggatttggcttcgaatctc | To prepare the yeast expression construct |
| TfSMT1-1-R | ACTAGTgttgctatctgaagcaggcttc |  |
| TfSMT1-2-F | GAATTCatggaacatcataagatcttcatatattt | To prepare the yeast expression construct |
| TfSMT1-2-R | ACTAGTgttgctatctgaagcaggcttc |  |
| AtCAS-F | aggagaaaaaaccccGGATCCatgtggaaactgaagatcgcg | To prepare the yeast expression construct |
| AtCAS-R | caacttctgttccatGTCGACtcattctccttgttgcaataatacc |  |
| Ox-TfSMT1-1-F | GGGGACAAGTTTGTACAAAAAAGCAGGCTGCCACCatggatttggcttcgaatctc | To prepare the plant overexpression construct |
| Ox-TfSMT1-1-R | GGGGACCACTTTGTACAAGAAAGCTGGGTAtcagttgctatctgaagcaggcttc |  |
| RNAi-TfSMT1-1-F | GGGGACAAGTTTGTACAAAAAAGCAGGCTGCCACCatgggttaataaatactatgatcttgcgac | To prepare the plant RNAi construct |
| RNAi-TfSMT1-1-R | GGGGACCACTTTGTACAAGAAAGCTGGGTAttatttaacactctataaatctctttgtagca |  |
| actin-F | TCGCTGCTGAGGTTTTGGAA | For the qRT-PCR analysis |
| actin-R | CCAATTTCGCCTTTGCCCTT |  |
| RT-TfSMT1-1-F | CTAACGACTGTTGGACGCCT |  |
| RT-TfSMT1-1-R | CTTCCGCGCCAAAAAGAAGT |  |
